# *In vivo* study on heparin/poly-l-lysine-copper coating for surface functionalization of ureteral stent

**DOI:** 10.1093/rb/rbac083

**Published:** 2022-10-21

**Authors:** Bukola O Awonusi, Jianzhong Li, Hongwei Li, Zhenyu Wang, Jiyuan Hu, Ke Yang, Jing Zhao

**Affiliations:** Shi-Changxu Innovation Center for Advanced Materials, Institute of Metal Research, Chinese Academy of Sciences, Shenyang 110016, China; School of Materials Science and Engineering, University of Science and Technology of China, Hefei 230026, China; Department of Urology, General Hospital of Northern Theater Command, Shenyang 110840, China; Department of Urology, General Hospital of Northern Theater Command, Shenyang 110840, China; Department of Urology, General Hospital of Northern Theater Command, Shenyang 110840, China; Department of Urology, The First Affiliated Hospital of China Medical University, Shenyang 110001, China; Shi-Changxu Innovation Center for Advanced Materials, Institute of Metal Research, Chinese Academy of Sciences, Shenyang 110016, China; Shi-Changxu Innovation Center for Advanced Materials, Institute of Metal Research, Chinese Academy of Sciences, Shenyang 110016, China

**Keywords:** ureteral stent, heparin/PLL-Cu nanoparticles, in vivo implantation, bacterial adhesion, encrustation

## Abstract

Polyurethane (PU) ureteral stents are used in clinics to maintain the ureteral patency. Due to biofilm formation and encrustation complications, long-term clinical usage has been limited. It is therefore necessary to develop an effective response to this unmet medical need. A heparin/poly-l-lysine/copper (NPs) coating was developed in our previous work that showed the effect of preventing infection and encrustation *in vitro*. In this work, a further study was conducted by grafting NPs on clinical ureteral stents that then were implanted into the infectious bladders of Wistar rats to investigate the effects of nanoparticles on bacterial growth and crystal deposition *in vivo*. It was found that decreased numbers of adherent microbes, urease amount splitting by bacteria, and deposited crystals were observed on the NPs stents with significant differences in comparison with PU stents. Besides, histological analysis showed that the NPs stents decreased the host tissue inflammation in close relation to the decrease biofilm formation and encrustation after 28 days of implantation.

## Graphical Abstract

**Figure:**
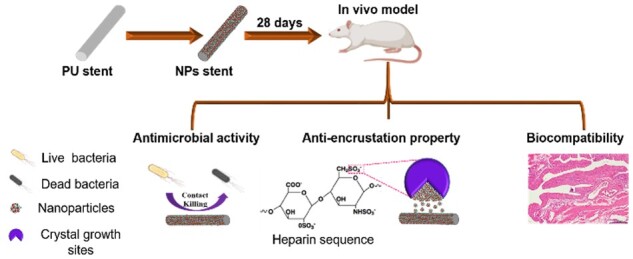

## Introduction

The urinary system, which plays an important role in urine drainage, is essential for the human body [1]. Ureteral stents made of polyurethanes (PUs) are widely applied due to their good biocompatibility, mechanical and physical properties [2]. It was used to aid the outflow of urine from the kidney to the bladder [3]. Nevertheless, these indwelling ureteral stents are beneficial in a variety of clinical situations. Due to the non-sterile nature of their applications, their use may result in severe complications such as infection, bacterial colonization and encrustation formation on stent surfaces.

Infections of the urinary tract (UTIs) caused by contamination of the ureteral stent as well as pain associated with secondary stent removal are major problems in treating urinary tract disorders. Due to continual contact with urine, the formation of bacterial biofilm and deposition of urinary crystals are significant problems [4]. Biofilm-forming bacteria are known to easily bind to ureteral stents within 24 h of implantation, and the accumulation of microorganisms results in harmful and severe complications associated with the use of implant materials [5, 6]. As a result of bacteria adhering to ureteral stents, intraluminal and extraluminal biofilms enables the entry and persistence of uropathogens in the bladder and thus enhance the development of resistance.

Furthermore, the deposition of mineral crystals can be aggravated attributing to the infection, which is associated with urease-positive bacteria secreting ammonia and carbon dioxide through the hydrolysis of urea in the urine, contributing to the elevation of urine pH, and the promotion of calcium and magnesium phosphate precipitation [7, 8]. Certain urinary tract pathogens such as *Proteus mirabilis, Escherichia coli* and *Pseudomonas aeruginosa* are known to promote crystal growth [9]. Sequentially, a continued occurrence of infection and encrustation leads to the obstruction of urine flow, hydronephrosis and even serious sepsis [10]. Due to these complications, a dual functional ureteral stent with antibacterial and anti-encrustation properties is significant in the field of urology.

Copper has been reported to kill bacteria by disrupting the bacteria cell membrane [11, 12]. Silva *et al.* [13] demonstrated that a copper coating inhibited the growth of *Staphylococcus aureus* after 10 min of direct contact between the bacteria and the coating surface. Meanwhile, glycosaminoglycans tend to decrease encrustation due to their binding ability to urinary components, which blocks the sites associated with crystal growth [14–16].

Accordingly, Hep/PLL-Cu (NPs) coating has been prepared for ureteral stent application in our previous work. The characterization of NPs, antimicrobial activity against *P. mirabilis* and the inhibitory effect of heparin nanoparticles on the formation of encrustation have been shown *in vitro* [17] compared to clinical PU. The NPs stent could inhibit bacteria and encrustation through the continuous release of Cu^2+^ and Hep from the nanoparticles. Based on our previous studies on anti-infection and anti-encrustation, the focus of this study was to further investigate the antibacterial and anti-encrustation abilities *in vivo* of NPs immobilized ureteral stents by evaluating the antibacterial performance, urease secretion, encrustation formation and histopathological response to tissues.

## Materials and methods

### Materials

PU ureteral stent was purchased from Reborn Medical Co., Ltd, China. Dopamine hydrochloride (DA-HCl), Tris base, toluidine blue O (TBO) and copper chloride (CuCl_2_) were obtained from Sigma–Aldrich. Heparin sodium (Hep, Mw<8 kDa) and poly-_L_-lysine (PLL, Mw 150–300 kDa) were purchased from Shanghai Bioscience and Technology Company, China. All the reagents were in analytical grade and were used without further purification.

### NPs immobilized stents (NPs stents) preparation

**Figure 1.**
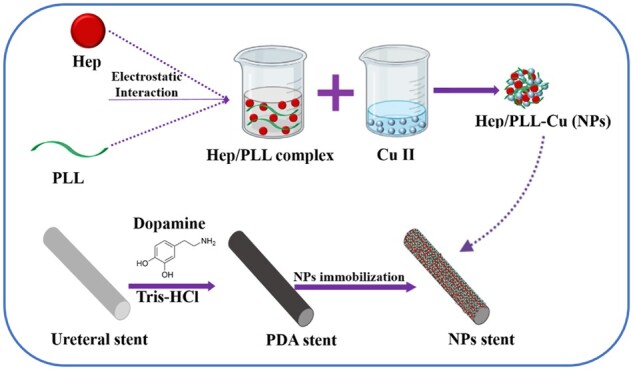
Schematic illustration of the immobilization method of NPs on the dopamine-coated PU.

A schematic drawing of NPs preparation and immobilization is shown in Fig. 1. PU stents were cut into 3 mm in length, followed by rinsing with water and ethanol for 10 min, respectively. Subsequently, 2 mg/ml dopamine solution was prepared using Tris buffer (pH = 8.5). Then, the cleaned PU stents were vertically immersed in dopamine solution and incubated for 24 h. After rinsing with deionized water and drying, the dopamine-coated stents were immersed in the NPs solution at 37°C for 24 h under mild shaking. The NPs solution was prepared by using a uniform volume of Hep solution (5 mg/ml) and PLL solution (1 mg/ml) mixture in an ultrasonic condition for 10 min, followed by adding a certain volume of CuCl_2_ solution (2 mg/ml). Finally, the resulting immobilized stents were rinsed with deionized water three times.

### Surface characterization

X-ray photoelectron spectroscopy (XPS, XSAM800, Kratos Ltd, UK) analysis on the stents was carried out with an Al Ka X-ray source (1486.6 *eV*) for elemental composition measurement. The surface of the coating was investigated by scanning electron microscope (SEM, JSM-6301 F, Japan) equipped with Energy-dispersive X-ray spectrometry (EDS, Oxford INCAX 7582, UK) to determine the elemental compositions of the coating.

### Animals and surgical procedures

The surgical procedure and all animal studies were conducted according to the approval of the institutional animal care and use committee (IACUC) in Liaoning Changsheng Biotechnology Co. Ltd (Approval No., CSE202109001). A total of 68 Wistar rats (8–10 weeks old, male) weighing between 300 and 330 g were selected and randomly divided into two groups, i.e. the coated group (NPs stents, 10 rats in each group) and the bare group (PU stents, seven rats in each group) at time intervals. The rats were placed in the supine position and general anesthesia was induced by intraperitoneal injection of sodium pentobarbital (30 mg/kg) before implantation. Then, a 3-mm long stent was inserted into the rat’s bladder. Simultaneously, 0.2 ml *P. mirabilis* at a concentration of 3 × 10^6^ CFU/ml was injected into the bladder and kept for 10 min. After being fed for 7, 14, 21 and 28 days, respectively, the indicated rats were sacrificed and stents were retrieved for further evaluation.

### Surface morphology observation

Observation on a scanning electron microscope (SEM, FEI 430 Nano, USA) was performed to analyze the encrustation morphology. Stents were rinsed with PBS to detangle the non-adherent tissues, then fixed with 2.5% glutaraldehyde and dehydrated in a series of ethanol. After drying for 48 h, stents were coated with a thin gold layer and then observed by SEM.

### Calcium and magnesium measurement

Three ureteral stent samples were sonicated in 5% HCl solution for 30 min to dissolve the deposited crystals (hydroxyapatite-Ca, struvite-Mg). Inductively coupled plasma optical emission spectrometry (ICP-OES, 5110, USA) was used to quantitatively measure the amount of calcium (Ca) and magnesium (Mg) present in the resulting solutions.

### Heparin release

The NPs stents were retrieved at specific time intervals and rinsed with PBS to detangle the non-adherent tissues. After that, the heparin release from the NPs stents at different time implantation was determined by a TBO assay. Briefly, each specific NPs stent was incubated with 4 ml TBO solution under a static condition at 37°C for 4 h and rinsed twice with deionized water. Subsequently, the Hep/TBO complex formed on the NPs stent was dissolved in a 5 ml ethanol/0.1 M NaOH mixture. Then, 150 µl of the supernatant was measured at 530 nm by a microplate reader (Quant, Bio-Rad, USA) and evaluated by a calibration standard curve. The amount of heparin released was determined by the following equation:

(1) $$\text{Heparin release}\;\left(\text{mg}/{\text{cm}}^{2}\right)=[Z_{1}–Z_{2}]/S,$$

where *Z*_1_ denotes the amount of heparin loaded on the stent before implantation, *Z*_2_ denotes the amount of heparin after stent removal at 7, 14, 21 and 28 days, and *S* denotes the outer surface area of the stent.

### Bacterial attachment measurement on stents

Quantification of the adherent bacteria on the stents was carried out by the plate count method. Three stents from each group at every time point were rinsed with PBS to remove non-adherents from the stent surface. All stents were aseptically transferred into tubes containing PBS and vortexed for 3 min. Serial dilutions of the resulting bacterial suspension were done and 100 µl of the dilution was cultured with nutrition agar for 24 h. After incubation, bacterial colonies were counted.

### Urine collection

Rats were kept in an aseptic metabolic cage for 24 h at the end of the 6th, 13th, 20th and 27th days for urine collection. After that, the urine was used to test bacteria colonies, pH value and urease secretion.

### Planktonic microbes and pH measurement

The plate counting method was used to test planktonic microbes in urine with serial dilutions and incubation. The pH values were measured by a pH meter (PHSJ-4F, Rex Electric Chemical).

### Urease secretion testing

The urine collected for 24 h was centrifuged at 3000 rpm for 20 min to remove the depositions. The supernatant liquid was used in the following test. Enzyme-linked immunosorbent assay (ELISA, Mlbio, China) was used to measure the level of urease secretion according to the manufacturer’s protocol. Fifty microliters of urine was diluted in 2-fold serial with 1% bovine serum albumin (BSA) in PBS and then incubated for 30 min at 37°C. After washing with wash buffer (0.05% Tween-20 in PBS) three times, 50 μl detection horseradish peroxidase (HRP)-labeled antibody was added to the plates and incubated for 30 min at 37°C. Then 100 μl 3,3′,5,5′-tetramethylbenzidine (TMB) was added and cultivated in dark for 10 min. Stop solution was added to each well, then the optical density was measured at 450 nm by a microplate reader (Bio-Rad, USA). The mean values of samples in triplicates were used to calculate the concentration with a range of 0.5–150 ng/ml and fitted by the standard.

### Histopathological observation

At the indicated times, rats were sacrificed for histopathological observation. The bladder was fixed in 4% formaldehyde and dehydrated in a series of ethanol, embedded in paraffin, which was then cut into slices with a thickness of 4 µm and fixed on glass slides. All slices were deparaffinized by xylene and dehydrated by a graded concentration of ethanol. After rinsing in distilled water, slices were stained with hematoxylin–eosin (H&E) dye for 3 min, rinsed with water, separated in 70% alcohol and stained in 0.01% eosin for 5 s. Lastly, the tissues were dehydrated with ethanol and rinsed with xylene. The slices were observed under an optical microscope (Olympus, BX46, Japan).

### Statistical analysis

The Student’s *t* was performed by Origin 9.1 software to evaluate the difference in data sets. Data were presented as mean±SD, *P* values <0.05 were expressed as statistically significant.

## Results

### Surface characterization

**Figure 2.**
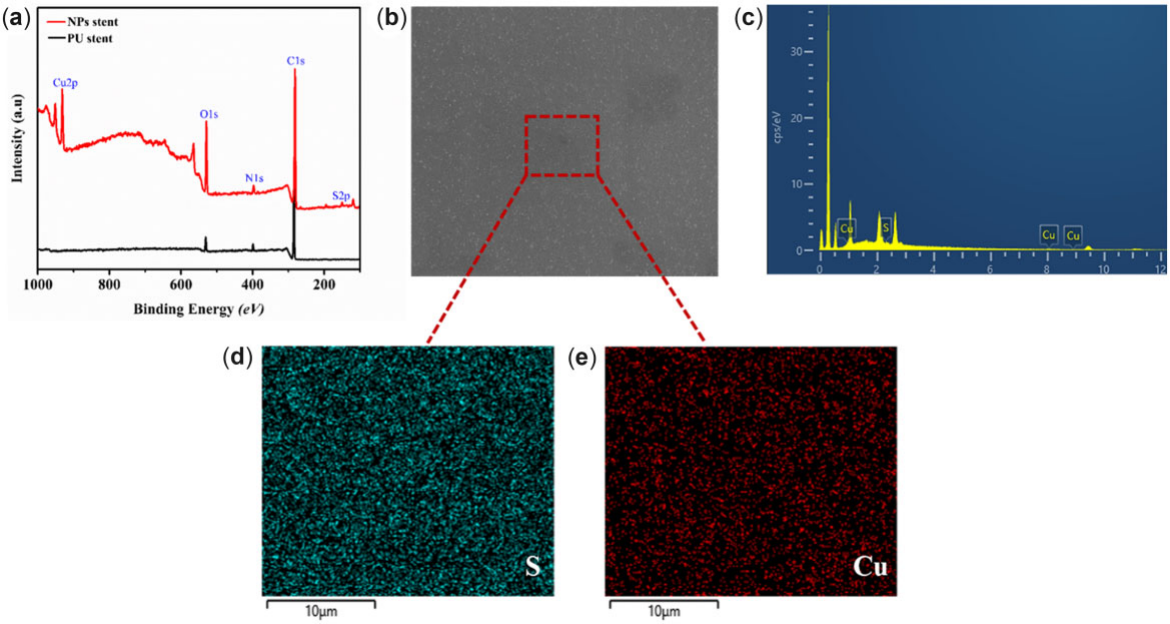
(**a**) XPS Wide scan of PU stent and NPs stent; (**b**) SEM micrograph of NPs stent; (**c**) EDS elemental spectrum from the red box in (**b**) of NPs stent; and (**d** and **e**) mapping analyses of sulfur and copper.

Figure 2a shows the XPS wide-scan spectra of both PU stent and NPs stent with respective surface elemental compositions. Evidently, the NPs altered the surface chemical composition by the presence of new S2p and Cu2p peaks. The S2p peak at 168.5 *eV* was from the characteristic sulfo group in heparin, and the Cu2p peak at 932.5 *eV* was from the copper grafted on samples, which was also confirmed by EDS spectrum (Fig. 2c). Figure 2b shows the SEM surface morphology of the NPs stent, and Fig. 2d and e depict the mapping analysis of the NPs coating surface, which showed a uniform distribution of the NPs.

### Surface morphology observation

**Figure 3.**
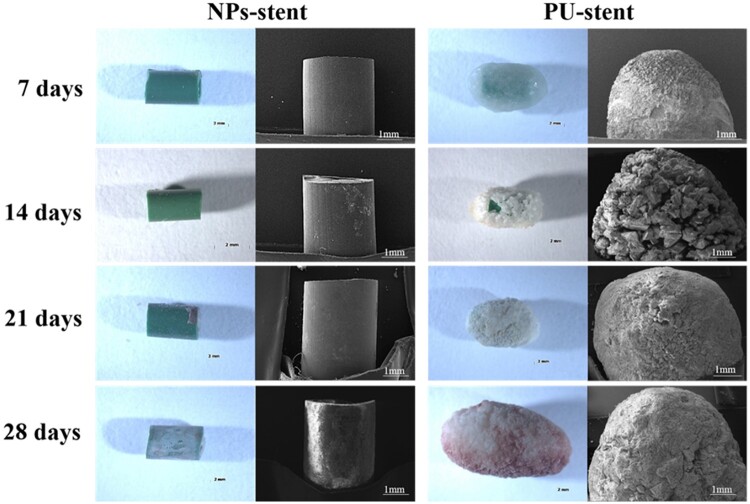
Photos and SEM images of ureteral stents after 7, 14, 21 and 28 days of implantation.

SEM micrographs of encrustation on NPs stents and PU stents are displayed in Fig. 3. On the 7th day, crystal deposits were evident on the surface of the PU stent. Whereas, there was no deposition on the surface of NPs stent. Similar results were shown on the 14th day, nevertheless, the PU stents were covered with crystals and the encrustation appeared larger. When implanted for 21 and 28 days, the encrusted crystals were significantly more and bigger on PU stents. While NPs stents revealed a dramatically reduced property of encrustation, indicating a promising ureter coating.

### Calcium and magnesium depositions

**Figure 4.**
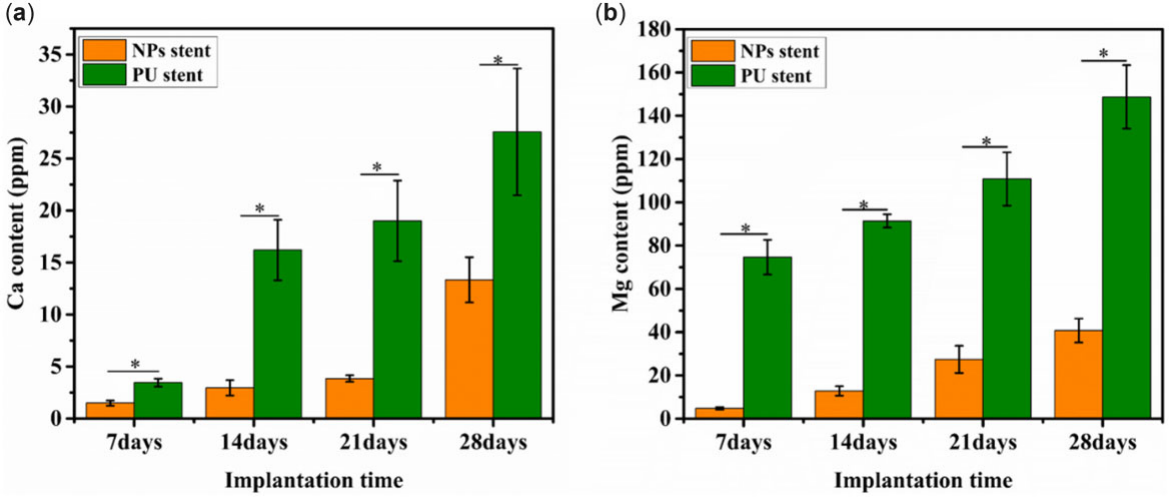
(**a**) Ca and (**b**) Mg contents in encrustation measured by ICP-OES for different implantation times. Data were presented as mean and SD. (*n* = 3, *P <* *0.05).

The encrustation levels on samples were quantitatively evaluated by ICP-OES for 7, 14, 21 and 28 days. The deposition amounts of Ca and Mg ions are displayed in Fig. 4a and b. However, for each implantation time, the highest levels of encrustation (in terms of both Ca and Mg) were measured on the bare PU stents, indicating that NPs stents provided resistance to encrustation.

### Heparin release

**Figure 5.**
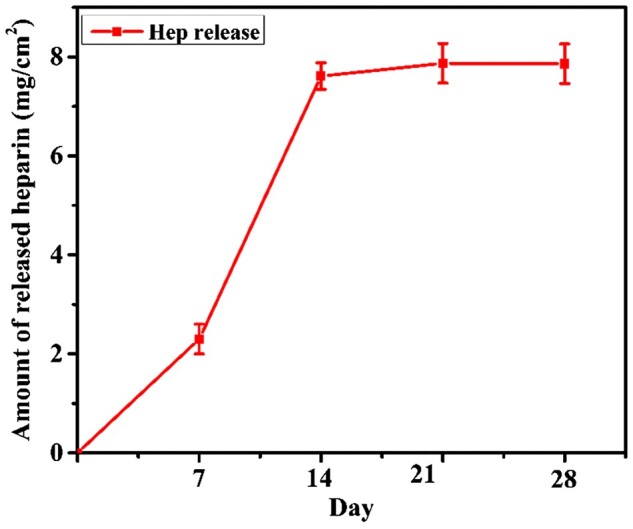
Heparin release from NPs stent. Data were presented as mean, *n* = 3.

The cumulative amount of released heparin at each time point was determined by the TBO assay. Figure 5 shows the release profile of heparin from NPs stent during each implantation time, and it can be observed that there was a slow-release rate in the first seven days, followed by a nearly constant release rate during the entire implantation.

### Bacterial attachment on stents and planktonic microbes in urine

**Figure 6.**
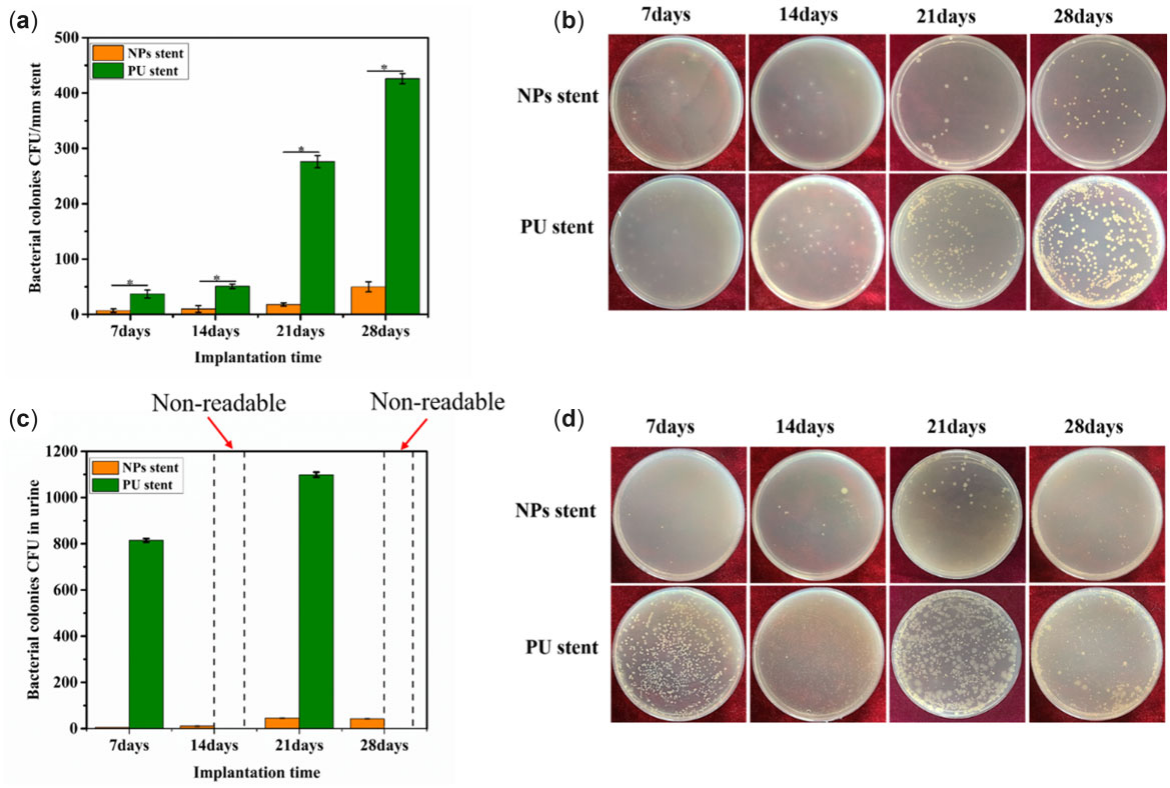
(**a**) Bacterial adherence on stents; (**b**) plates photos of stents for 7, 14, 21 and 28 days; (**c**) planktonic microbes in urine; and (**d**) bacteria growth on plates. Data were presented as mean and SD. (*n* = 3, *P <* *0.05).

Planktonic microbes in urine and the total number of bacterial growths on the surfaces of NPs stents and PU stents were evaluated using the plate count method. Figure 6 depicts the bacterial growth on the stents cultured in urine for 7, 14, 21 and 28 days. In contrast to the PU stent, the bacteria adhered to the NPs stents were significantly fewer for each indwelling time. Also, the urine bacterial culture results appeared too many, which were non-readable.

### pH of urine

**Figure 7.**
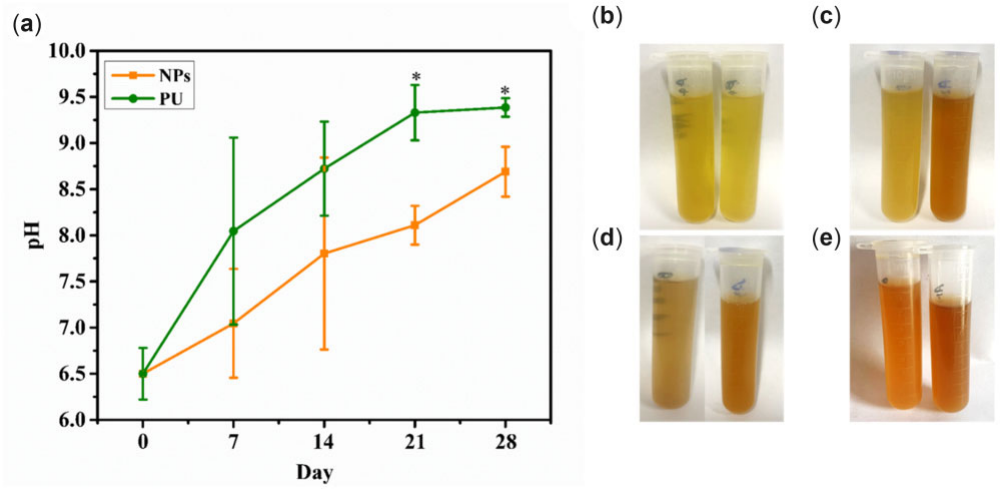
(**a**) pH curves of animal urine collection for 24 h; photos of urine for 7 days (**b**), 14 days (**c**), 21 days (**d**) and 28 days (**e**), left (NPs stents), right (PU stents). Data were presented as mean and SD. (*n* = 3, *P <* *0.05).

The pH of animal urine was monitored and measured at different time intervals, as plotted in Fig. 7a. It can be observed that the pH of urine from the bladder with NPs stent was lower (8.69 ± 0.27) than that with PU stent (9.38 ± 0.1), which illustrates that the urease activity of *P. mirabilis* could increase the pH of urine and thus enhance the encrustation own to a higher alkaline environment. Moreover, the changes in pH caused the urinary discoloration in the urine from the bladder with PU stent, which was a result of infection, as presented in Fig. 7b–e.

### Urease secretion

**Figure 8.**
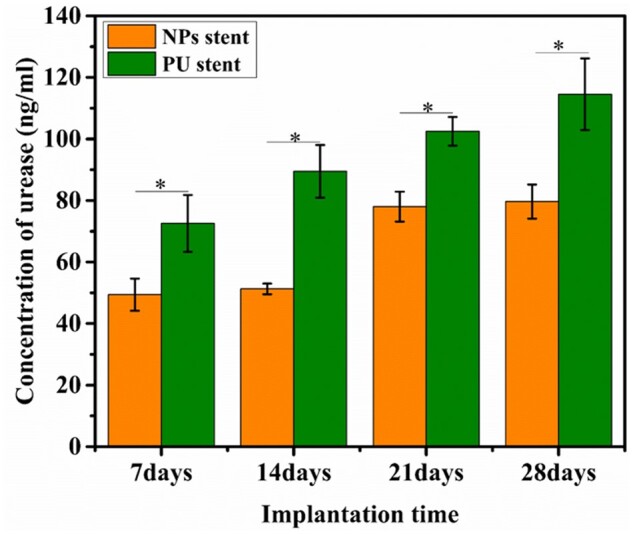
Amounts of urease secreted after co-culturing with NPs stent and PU stent, respectively. Data were presented as mean and SD. (*n* = 3, *P <* *0.05).

To confirm whether the elevation of pH was induced by urease secretion from *P. mirabilis*, the urease discharge activities of urine incubated with NPs stent and PU stent were detected and are depicted in Fig. 8. After 7 and 14 days of incubation periods, the amounts of urease released from NPs stent group were 45.61 and 50.65 ng/ml, respectively, which were lower than those from PU stent group, 70.8 and 99.2 ng/ml. On Days 21 and 28, it was observed that the amount of urease released from NPs stent group was significantly lesser than that from PU stent group.

### Histopathological analysis

**Figure 9.**
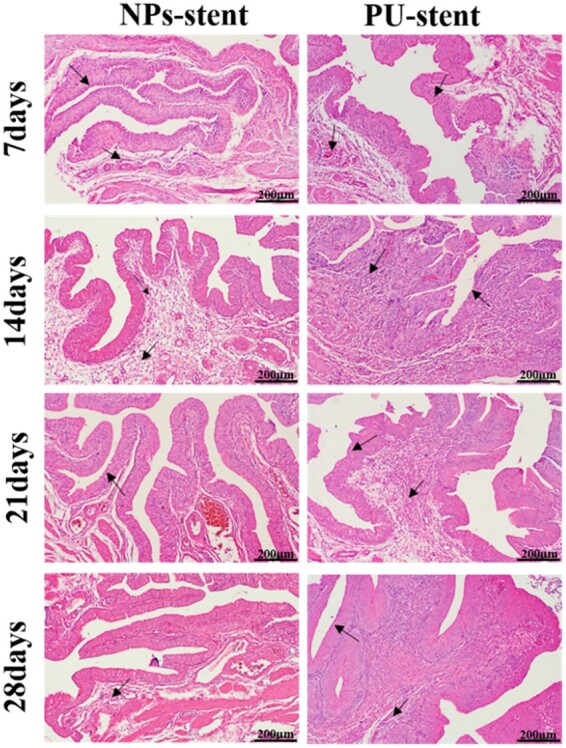
Representative H&E-stained bladder sections at 7-, 14-, 21- and 28-day post-implantation, with arrows pointing to the cell infiltration.

H&E staining micrographs presented no severe inflammation of NPs stents in the period of implantation, as depicted in Fig. 9. It can be observed that on Day 7, NPs stents showed almost no inflammation of tissues. Whereas, infiltration of mononuclear cells along with epithelial degeneration was significantly observed on the PU stents on Days 14 and 21. Meanwhile, limited mononuclear cell infiltration was detected on NPs stents. On Day 28, histopathological micrographs of NPs stent showed limited inflammation, whereas, PU stent revealed intense inflammation.

## Discussion

Among the most common hospital-acquired infections, urinary tract infections tend to cause the greatest morbidity in patients. Recurrently, the ureteral stent-associated infection may result in urosepsis and death with long-term insertion [18]. Sadly oral antibiotics alone may prevent the growth of planktonic bacteria in the urinary tract, but it has not been observed to have a significant effect on bacterial growth directly on the intervention stents. In light of this, surface modification of urinary catheters with broad-spectrum antibacterial agents has been reported to inhibit biofilm formation [19]. Besides, encrustation on stents that blocks the lumen is another critical factor in the occurrence of urinary tract infections [20]. Recently our previous studies [17] showed that the Hep/PLL-Cu (NPs) coating as a surface modification on PU could effectively inhibit microbe’s attachment and resist encrustation *in vitro*. Accordingly, in this present study, further investigation on the anti-encrustation property of NPs as a ureteral stent coating was conducted *in vivo* by studies of antibacterial performance, urease secretion and encrustation formation. The NPs in Tris buffer were formed by the electrostatic interaction between negatively charged Hep and positively charged PLL coupled with Cu ions. Successful immobilization of NPs on the stent was demonstrated in Fig. 2. From the XPS spectra, there were two new peaks: S2p (168.5 *eV*) and Cu2p (932.5 *eV*) identified on the NPs spectrum. The S2p peak indicated a successful immobilization of heparin by the presence of the sulfate group (${\text{SO}}_{3}^{-}$) in the heparin backbone [21], while the Cu2p confirmed the presence of copper. Furthermore, the SEM and EDS results demonstrated a uniform distribution of the NPs.

Bultitude *et al.* [22] reported that the incidence rates of encrustation deposition on double J stents were 45% and 75.5% within 4 and 6 months, respectively, which revealed that the tendency of encrustation over a double J stent increased with increased indwelling time. Figure 3 reveals the crystal depositions on the NPs and PU stents with the implantation time of the stent. It was observed that throughout the implantation time, PU stents showed a large amount of encrustation in comparison to the NPs stents. Besides infection, the blockage of the ureteral stent by encrustation remains a major problem with a significant impact on the quality of a patient’s life, which would cause painful distention of the bladder or kidney stones. The presence of ammonia generated by bacterial urease enzyme [20] leads to the precipitation of Ca and Mg. Ca and Mg ions are soluble products of hydroxyapatite and struvite, which play a major role in encrustation formation [23]. Amounts of Ca and Mg deposits were evaluated quantitatively using ICP-OES, as shown in Fig. 4. The Ca and Mg contents on the NPs stents revealed lesser amounts than the PU stents, indicating the effect of the nanoparticles in crystal growth reduction. It has been confirmed that heparin, which is a type of glycosaminoglycans, has the potential to inhibit the encrustation by acting as a crystal growth inhibitor when the sulfate group and uronic acid in the heparin molecule bind to the urine components [16, 24]. The anti-encrustation mechanism of our NPs coatings was mainly due to the release of heparin from the NPs, and as shown in Fig. 5, the amount of encrustation was reduced. Besides, for encrustation to occur on ureteral stents, infection is the most critical and irreversible factor [25].

The use of implant materials for urinary drainage is steadily increasing in urology. However, bacterial growths, as well as implant related infection, are major concerns [26]. Various pathogens present at the site of stent insertion or in urine quickly attach to the stent surface and colonize progressively to form a mature biofilm. *Proteus mirabilis,* a leading uropathogen, causes complicated urinary tract infections (UTIs) through the action of its urease enzyme, which decomposes urea into ammonia and carbon dioxide contributing to urine alkalization [27]. Thus, the bacterial growth on stents cultured in urine was evaluated by the plate counting method. At every time point, significantly fewer bacteria adhered to the NPs stent than the PU stent, as depicted in Fig. 6. The main reason for the antibacterial ability of the NPs stent was the continuous release of Cu ions from the surface of NPs stent, which affects the bacterial cytomembranes. As a result of the Cu ions released from the NPs, the amount of urease produced by *P. mirabilis* was reduced, preventing the elevation of urine pH and the precipitation of Ca and Mg phosphate crystals on the stents [28]. Urease, a characteristic bacterial enzyme is known to play a crucial role in the pathogenesis of *P. mirabilis* infection [29, 30]. The urease produced by *P. mirabilis* decomposes urea to produce ammonia and carbon dioxide resulting in an alkaline urine level. Figure 7 shows that the initial urine pH was 6.50, but due to the urease activity of *P. mirabilis,* an increase in the pH value of urine was observed over time. However, the pH value of urine cultured with the NPs stent reached 8.5, while that with the PU stent was significantly higher, which could induce more crystal precipitation. Meanwhile, the amount of urease released by the NPs stent group was lesser than the PU stent group, as depicted in Fig. 8. Therefore, since the urease activity of *P. mirabilis* increases the pH of urine, it appears that the increase of urine pH promotes the encrustation.

Although the above results have proven significant resistance to bacterial attachment and encrustation of the NPs stents, it is still important to investigate the biocompatibility of these ureteral stents. It has been demonstrated that the composition and crystallization behavior of rat urine is similar to that of human urine [31, 32]. The Wistar rats were dissected and the bladder showed no obvious abnormalities after implanting the NPs stents. In Fig. 9, the rat bladder samples with NPs stents showed less degree of inflammation than the PU stents at each implantation time. In addition, the degree of inflammation increased in the PU stent group on Day 28. The difference in the pathological results between the NPs stent and PU stent would most likely depend on different encrustation amounts on stents. The inflammation in the PU stent group was mainly caused by the amount of encrustation, which would irritate the bladder. Furthermore, the results from the NPs stents revealed that there was no toxic effect on ureteral epithelium cells, even though there was a continuous release of Cu ions and heparin particles from the stents. This validated the feasibility and functionalization of our NPs stents for ureteral application.

## Conclusion

In this *in vivo* study, we further proved the potential efficacy of Hep/PLL-Cu (NPs) as an ideal coating on ureteral stents in relieving infection and encrustation formation compared to the clinically used PU stents. The NPs stents demonstrated significant antibacterial properties both on the stent’s surface and in urine. Moreover, the infectious encrustation deposits (Ca and Mg) were significantly reduced on the surface of NPs stents in the presence of microorganisms compared to PU stents due to the continuous release of Cu^2+^ ions and heparin. The NPs stents could obviously reduce urease production, resulting in a lower pH value of infected urine. In addition, the NPs stents displayed less inflammation in the host tissue due to the absence of cytotoxicity, bacterial and encrustation formation during the 28 days of implantation. Overall, the properties exhibited by the NPs stent suggest its potential use as a urinary system medical device.

## Funding

Liaoning Science and Technology Program (grant No.2020JH2/10300159).

*Conflicts of interest statement*. None declared.

## References

[1] Öztürk R , Murt A. Epidemiology of urological infections: a global burden. World J Urol 2020;38:2669–79. 31925549 10.1007/s00345-019-03071-4

[2] Wang Y , Hong Q, Chen Y, Lian X, Xiong Y. Surface properties of polyurethanes modified by bioactive polysaccharide-based polyelectrolyte multilayers. Colloids Surf B Biointerfaces 2012;100:77–83. 22771524 10.1016/j.colsurfb.2012.05.030

[3] Mosayyebi A , Manes C, Carugo D, Somani BK. Advances in ureteral stent design and materials. Curr Urol Rep 2018;19:1–9. 29637309 10.1007/s11934-018-0779-y PMC5893657

[4] Zumstein V , Betschart P, Albrich WC, Buhmann MT, Ren Q, Schmid HP, Dominik A. Biofilm formation on ureteral stents – incidence, clinical impact, and prevention. Swiss Med Wkly 2017;147:0506. 10.4414/smw.2017.14408 28165539

[5] Reid G , Tieszer C, Denstedt J, Kingston D. Examination of bacterial and encrustation deposition on ureteral stents of differing surface properties, after indwelling in humans. Colloids Surf B 1995;5:171–9.

[6] Campoccia D , Montanaro L, Arciola CR. A review of the clinical implications of anti-infective biomaterials and infection-resistant surfaces. Biomater Sci 2013;34:8018–29. 10.1016/j.biomaterials.2013.07.048 23932292

[7] Lange D , Bidnur S, Hoag N, Chew BH. Ureteral stent-associated complications—where we are and where we are going. Nat Rev Urol 2015;12:17–25. 25534997 10.1038/nrurol.2014.340

[8] Broomfield RJ , Morgan SD, Khan A, Stickler DJ. Crystalline bacterial biofilm formation on urinary catheters by urease-producing urinary tract pathogens: a simple method of control. J Med Microbiol 2009;58:1367–75. 19556373 10.1099/jmm.0.012419-0

[9] Buhmann MT , Abt D, Nolte O, Neu TR, Strempel S, Albrich WC, Betschart P, Zumstein V, Neels A, Maniura-Weber K, Ren Q. Encrustations on ureteral stents from patients without urinary tract infection reveal distinct urotypes and a low bacterial load. Microbiome 2019;7:1–17. 30981280 10.1186/s40168-019-0674-x PMC6462311

[10] Gorman SP , Jones DS, Bonner MC, Akay M, Keane PF. Mechanical performance of polyurethane ureteral stents in vitro and ex vivo. Biomaterials 1997;18:1379–83. 9363338 10.1016/s0142-9612(97)00070-7

[11] Arendsen LP , Thakar R, Sultan AH. The use of copper as an antimicrobial agent in health care, including obstetrics and gynecology. Clin Microbiol Rev 2019;32:00125–18. 10.1128/CMR.00125-18 PMC6730497 31413046

[12] Mahmoodi S , Elmi A, Hallaj NS. Copper nanoparticles as antibacterial agents. J Mol Pharm Org Process Res 2018;6:1–7.

[13] da Silva FS , Cinca N, Dosta S, Cano IG, Guilemany JM, Caires CSA, Lima AR, Silva CM, Oliveira SL, Caires ARL, Benedetti AV. Corrosion resistance and antibacterial properties of copper coating deposited by cold gas spray. Surf Coat Technol 2019;361:292–301.

[14] Hildebrandt P , Sayyad M, Rzany A, Schaldach M, Seiter H. Prevention of surface encrustation of urological implants by coating with inhibitors. Biomaterials 2001;22:503–7. 11214762 10.1016/s0142-9612(00)00217-9

[15] Yoshimura K , Yoshioka T, Miyake O, Honda M, Yamaguchi S, Koide T, Okuyama A. Glycosaminoglycans in crystal‐surface binding substances and their role in calcium oxalate crystal growth. Br J Urol 1997;80:64–8. 9240182 10.1046/j.1464-410x.1997.00189.x

[16] Cauda F , Cauda V, Fiori C, Onida B, Garrone E. Heparin coating on ureteral double J stents prevents encrustations: an in vivo case study. J Endourol 2008;22:465–72. 18307380 10.1089/end.2007.0218

[17] Awonusi BO , Li J, Li H, Wang Z, Yang K, Zhao J. In vitro and in vivo studies on bacteria and encrustation resistance of heparin/poly-L-lysine-Cu nanoparticles coating mediated by PDA for ureteral stent application. Regen Biomater 2022;9:rbac047. 35928999 10.1093/rb/rbac047 PMC9345062

[18] Anjum S , Singh S, Benedicte L, Roger P, Panigrahi M, Gupta B. Biomodification strategies for the development of antimicrobial urinary catheters: overview and advances. Glob Chall 2018;2:1700068. 31565299 10.1002/gch2.201700068 PMC6607219

[19] Lange D , Chew BH. Update on ureteral stent technology. Ther Adv Urol 2009;1:143–8. 21789062 10.1177/1756287209341306 PMC3126057

[20] Sabbuba NA , Mahenthiralingam E, Stickler DJ. Molecular epidemiology of *Proteus mirabilis* infections of the catheterized urinary tract. J Clin Microbiol 2003;41:4961–5. 14605124 10.1128/JCM.41.11.4961-4965.2003 PMC262497

[21] Lee Y , Le TP, Seon GM, Ryu SB, Brophy CM, Kim Y, Park JC, Park KD, Cheung FJ, Sung HJ. Heparin-functionalized polymer graft surface eluting MK2 inhibitory peptide to improve hemocompatibility and anti-neointimal activity. J Control Release 2017;266:321–30. 28987880 10.1016/j.jconrel.2017.10.002 PMC5723561

[22] Bultitude MF , Tiptaft RC, Glass JM, Dasgupta P. Management of encrusted ureteral stents impacted in upper tract. J Urol 2003;62:622–6. 10.1016/s0090-4295(03)00506-5 14550429

[23] Gultekinoglu M , Kurum B, Karahan S, Kart D, Sagiroglu M, Ertaş N, Ozen AH, Ulubayram K. Polyethyleneimine brushes effectively inhibit encrustation on polyurethane ureteral stents both in dynamic bioreactor and in vivo. Mater Sci Eng C 2017;71:1166–74. 10.1016/j.msec.2016.11.125 27987673

[24] Riedl CR , Witkowski M, Plas E, Pflueger H. Heparin coating reduces encrustation of ureteral stents: a preliminary report. Int J Antimicrob Agents 2002;19:507–10. 12135841 10.1016/s0924-8579(02)00097-3

[25] Zhao J , Ren L, Zhang B, Cao Z, Yang K. In vitro study on infectious ureteral encrustation resistance of Cu-bearing stainless steel. J Mater Sci Technol 2017;33:1604–9.

[26] Tenke P , Riedl CR, Jones GL, Williams GJ, Stickler D, Nagy E. Bacterial biofilm formation on urologic devices and heparin coating as preventive strategy. Int J Antimicrob Agents 2004;23:67–74. 15037330 10.1016/j.ijantimicag.2003.12.007

[27] Yuan F , Huang Z, Yang T, Wang G, Li P, Yang B, Li J. Pathogenesis of *Proteus mirabilis* in catheter-associated urinary tract infections. Urol Int 2021;105:354–61. 33691318 10.1159/000514097

[28] Heidari ZH , Juhart V, Vass A, Franz G, Jocham D. Efficacy of silver/hydrophilic poly (p-xylylene) on preventing bacterial growth and biofilm formation in urinary catheters. Biointerphases 2017;12:011001. 28100054 10.1116/1.4974197

[29] Milo S , Heylen RA, Glancy J, Williams GT, Patenall BL, Hathaway HJ, Thet NT, Allinson SL, Laabei M, Jenkins ATA. A small-molecular inhibitor against *Proteus mirabilis* urease to treat catheter-associated urinary tract infections. Sci Rep 2021;11:1–15. 33580163 10.1038/s41598-021-83257-2 PMC7881204

[30] Trotter J , McCoy C, Irwin N, Gorman S, Carson L. The exploitation of urease-producing bacteria in catheter-associated urinary tract infections using an infection-responsive biomaterial. World Biomaterials Congress, Montreal, UK: Queen's University, Belfast. 2016.

[31] Atmani F , Khan SR. Characterization of uronic-acid-rich inhibitor of calcium oxalate crystallization isolated from rat urine. Urol Res 1995;23:95–101. 7676539 10.1007/BF00307939

[32] Miyake O , Yoshioka T, Yoshimura K, Honda M, Yamaguchi S, Koide T, Okuyama A. Expression of Tamm-Horsfall protein in stone-forming rat models. Br J Urol 1998;81:14–9. 9467470 10.1046/j.1464-410x.1998.00493.x

